# Spirometry in chronic obstructive pulmonary disease in Norwegian general practice

**DOI:** 10.1186/s12875-020-01310-x

**Published:** 2020-11-18

**Authors:** Mette C. Tollånes, Geir E. Sjaastad, Bernt B. Aarli, Sverre Sandberg

**Affiliations:** 1 Norwegian Organization for Quality Improvement of Laboratory Examinations, Haraldsplass Deaconess Hospital, Bergen, Norway; 2 Holter legekontor, 2034 Holter, Norway; 3 Department of Clinical Science, University of Bergen, Bergen, Norway; 4 Department of Thoracic Medicine, Haukeland University Hospital, Bergen, Norway; 5 Department of Global Public Health and Primary Care, University of Bergen, Bergen, Norway; 6 Department of Medical Biochemistry and Pharmacology, Haukeland University Hospital, Bergen, Norway

**Keywords:** Spirometry, General practice, Chronic obstructive pulmonary disease, Web-based survey

## Abstract

**Background:**

General practitioners (GPs) in Norway increasingly use spirometry diagnostically as well as in follow up of patients with respiratory complaints, but little is known about their skills and knowledge in this area. The aim of the present study was to investigate how GPs interpret a case history and spirometry recordings of a patient with chronic obstructive pulmonary disease (COPD), and their knowledge about their own spirometer.

**Methods:**

A web-based survey, consisting of a case history and spirometry recordings of a patient with COPD, was distributed to the 4700 members of the Norwegian GP Association. In addition to background information about themselves and their spirometer, topics included whether they requested, and how they interpreted, a spirometry reversibility-test, identification of the of most likely diagnosis, and recognition of the spirometry parameters used to diagnose COPD and grade airway obstruction. Immediate feedback was provided for educational purposes.

**Results:**

Six hundred thirty GPs responded. Twenty six percent would not request a reversibility test, but 81% identified COPD as the most likely diagnosis. Less than 50% correctly identified the spirometry parameters used for diagnosis of COPD and grading the airway obstruction. One in five (21%) did not know which spirometer was used in their own practice, and 49 and 61% did not know which reference values were used for adults and children, respectively. Participants evaluated the survey as useful (average 74 points on a 0–100 scale) and would like more case-based surveys concerning use of spirometry in the future (average 91 points).

**Conclusion:**

In this cohort of self-selected GPs, probably more interested in respiratory medicine than the average GP, we identified several problem areas and gaps in knowledge regarding the use of spirometry.

**Supplementary Information:**

The online version contains supplementary material available at 10.1186/s12875-020-01310-x.

## Background

General practitioners (GPs) in Norway increasingly use spirometry diagnostically as well as a tool to evaluate lung function during follow up of patients with respiratory complaints. In 2018, approximately 97% of practicing GPs were reimbursed for performing spirometry tests, on average 30 times, with a total cost of approximately 41.9 million Norwegian kroner [1].

Previous studies have found that GPs do not always know when to request a spirometry, nor how to interpret it. In a study from seven Norwegian GP offices during 2009/2010, patients with a record of asthma or chronic obstructive pulmonary disease (COPD) were invited for clinical examination and spirometry to validate their diagnoses [2]. Of the 128 patients registered with COPD, only 74% fulfilled the spirometric criteria. Among the patients clinically diagnosed with asthma, 17% were re-diagnosed with COPD. Similar results were found in a UK study from 2017, where spirometries performed in primary care were re-assessed by pulmonologists [3]. While 96% of spirometry recordings were of adequate technical quality, only 73% of spirometries from patients with a clinical diagnosis of COPD were consistent with obstruction. A recent case-based survey among 250 Swedish GPs concluded that although the GPs indicated they would often request a spirometry when appropriate, only 23% could adequately interpret the spirometric recordings [4].

The Norwegian Organization for Quality Improvement of Laboratory Examinations (Noklus) has provided quality systems for point-of-care laboratory testing in primary care since 1992 [5], including courses, site visits, laboratory instrument evaluations, and external quality assurance (EQA) schemes. The main aim of the present study, conducted in collaboration with the Norwegian GP Association and the Norwegian Respiratory Society, was to investigate how GPs in Norway interpret a case history and spirometry recordings of a patient with COPD. A secondary aim was to investigate the GPs’ knowledge about their own spirometer.

## Methods

In 2019, we distributed a web-based multiple-choice survey (Additional file 1) to the 4712 practicing GPs registered as members of the Norwegian GP Association, with the following case history:

*A previously healthy man in the age group 50-59 years presents with complaints of progressive breathlessness on physical exertion over the past year. He has never had atopic symptoms, asthma or allergy, and there is no family history of lung- or upper airway disorders. He started smoking in his late teens but quit four years ago. You request a spirometry. Do you include a reversibility test or not?*

We asked which type of spirometer participants used (see Additional file 1 for details), and pictures of spirometry recordings were adapted accordingly to ensure recognizability. Participants were asked for the most likely diagnosis, and which spirometric parameters they used to diagnose the disease and grade airway obstruction. After choosing an answer, participants were immediately presented with the correct/preferred answer, as well as updated references for further reading. We also asked about general knowledge of their spirometer (need for calibration, reference values used), and some background information (geographic region, number of patients served, number of spirometries usually requested during a regular work week). Finally, we asked how useful they found the survey and if they would like more surveys concerning spirometry in the future.

The case history and spirometry recordings were of a patient with COPD, which was the correct most likely diagnosis. In line with the Norwegian Directorate of Health’s guidelines on management of COPD [6], we used the Global Initiative for Chronic Obstructive Lung Disease (GOLD) criteria of postbronchodilator *FEV*_*1*_*/FVC ratio* < 0.70 for diagnosis of COPD and *FEV*_*1*_ *in % of predicted* to grade the airway obstruction (≥ 80%: GOLD 1 – mild airway obstruction, 50–79%: GOLD 2 – moderate airway obstruction, 30–49%: GOLD 3 – severe airway obstruction, and < 30%: GOLD 4 - very severe airway obstruction) [7].

The survey was open exclusively to invited GPs for a four-week period, and only responses collected during this period were analyzed for the present study. Afterwards, a link to the survey was posted on Noklus’ website [8]. Descriptive statistics were performed using R version 3.6.1 [9]. Chi-square tests were used to investigate differences between groups, and *p*-values < 0.05 were considered statistically significant.

## Results

**Fig. 1:**
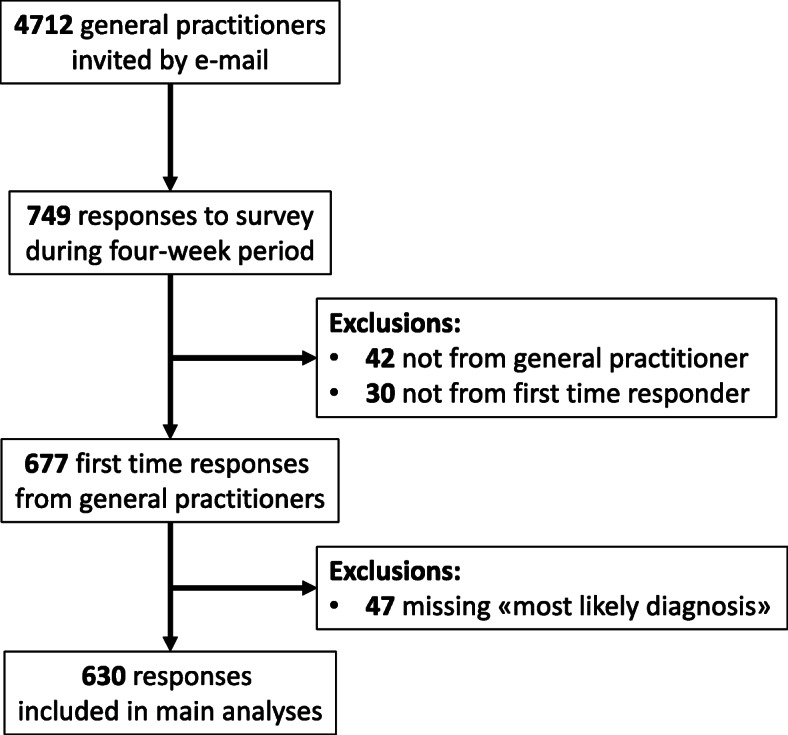
Flow chart of participants

A total of 749 responses were collected during the four-week period of data collection, partially or completely. Participants spent on average 5–6 min on the survey. Forty-two respondents indicated they were not currently practicing GPs. Of the remaining, 47 responses were excluded because respondents had not answered the question of the most likely diagnosis, and 30 because they were not first-time responses (Fig. 1).

Four hundred sixty-seven participants (74%) indicated they would request a reversibility test (Table 1), which was considered the correct answer, since this was the only option providing a post bronchodilator spirometry which is required to diagnose COPD [6]. When presented with pre- and postbronchodilator spirometry recordings, 582 (92%) correctly recognized that there was no significant reversibility (Table 1). Five hundred twelve participants (81%) correctly identified COPD as the most likely diagnosis. Only 287 (46%) correctly indicated they used only *FEV*_*1*_*/FVC ratio* for diagnosing COPD, and 269 (43%) correctly indicated they used only *FEV*_*1*_ *in % of predicted* to grade the airway obstruction.

**Table 1:** Distribution of responses to survey

Question	N responses (%)
Do you request a reversibility test?	
**Yes**	**467 (74.1)**
No	160 (25.4)
Missing	3 (0.5)
Is there significant reversibility?	
Yes	25 (4.0)
**No**	**582 (92.4)**
Don’t know	17 (2.7)
Missing	6 (1.0)
What is the most likely diagnosis?	
No pulmonary disorder/healthy	18 (2.9)
Asthma	9 (1.4)
Restrictive pulmonary disorder	26 (8.9)
**COPD**	**512 (81.3)**
Other	10 (1.6)
Don’t know	25 (4.0)
Which parameter(s) did you use to diagnose COPD?	
FVC	6 (1.0)
FVC in percent of predicted	11 (1.8)
FEV1	16 (2.5)
FEV1 in percent of predicted	32 (5.1)
**FEV1/FVC ratio**	**287 (45.6)**
FEV1/FVC ratio in percent of predicted	48 (7.6)
More than one option chosen	219 (34.8)
Missing	11 (1.8)
Which parameter(s) did you use to grade airway obstruction?	
FVC	7 (1.1)
FVC in percent of predicted	19 (3.0)
FEV1	113 (17.9)
**FEV1 in percent of predicted**	**269 (42.7)**
FEV1/FVC ratio	75 (11.9)
FEV1/FVC ratio in percent of predicted	42 (6.7)
More than one option chosen	59 (9.4)
Missing	46 (7.3)

Welch-Allyn (Hill-rom Services Inc) and Spirare (Diagnostica AS) were by far the most commonly used spirometers, but 133 participants (21%) did not know which type of spirometer was used in their practice (these participants were provided with Spirare printouts in the survey). The majority of participants indicated they would request between one and four spirometries per week, while 70 (11%) said they would request no spirometries during a regular working week (Table 2). GPs who requested more spirometries per week or served more patients, were more likely to recognize COPD as the correct diagnosis (*p*-values for trend < 0.001 and 0.02, respectively), but these factors were not associated with other outcomes. Type of spirometer used was generally not associated with performance in the survey (Table 2).

**Table 2:** Performance in survey (number (and fraction) of correct answers) by type of spirometer used, number of spirometries requested during a regular week, and number of patients served by the GP

	Requested reversibility test	Recognized non-sign. Reversibility test	Suggested COPD as likely diagnosis	Identified parameter ***FEV***_***1***_***/FVC ratio***	Identified parameter ***FEV1***_***1***_ ***in % of predicted***
**Type of spirometer used**					
Welch-Allyn (*n* = 311)	231 (0.75)	288 (0.93)	252 (0.81)	167 (0.54)	124 (0.40)
Spirare (*n* = 169)	129 (0.76)	159 (0.94)	143 (0.85)	69 (0.41)	80 (0.47)
Other/Don’t know (*n* = 150)	107 (0.72)	135 (0.90)	117 (0.78)	51 (0.34)	65 (0.43)
*p*-value for difference, all groups	0.65	0.38	0.32	< 0.001	0.28
*p*-value for difference, excluding “Other/Don’t know”	0.70	0.54	0.33	0.01	0.11
**Number of spirometries requested per week**					
None (*n* = 70)	53 (0.76)	64 (0.91)	48 (0.69)	32 (0.46)	29 (0.41)
1–4 (*n* = 403)	308 (0.77)	383 (0.95)	358 (0.89)	193 (0.48)	203 (0.50)
5 or more (*n* = 30)	27 (0.90)	29 (0.97)	29 (0.97)	19 (0.63)	15 (0.50)
Missing (*n* = 127)	79 (0.63)	106 (0.84)	77 (0.61)	43 (0.34)	22 (0.17)
*p*-value for difference, all groups	< 0.01	< 0.001	< 0.001	0.01	< 0.001
*p*-value for trend, excluding “Missing”	0.23	0.20	< 0.001	0.20	0.25
**Number of patients served by GP**					
< 1000 (*n* = 181)	142 (0.79)	167 (0.92)	148 (0.82)	88 (0.49)	93 (0.51)
1000–1499 (*n* = 294)	221 (0.75)	284 (0.97)	259 (0.89)	142 (0.48)	147 (0.50)
≥ 1500 (*n* = 33)	27 (0.82)	33 (1.00)	31 (0.94)	17 (0.52)	11 (0.33)
Missing (*n* = 122)	77 (0.64)	98 (0.80)	74 (0.61)	40 (0.33)	18 (0.15)
*p*-value for difference, all groups	0.02	< 0.001	< 0.001	0.02	< 0.001
*p*-value for trend, excluding “Missing”	0.88	0.75	0.02	0.67	0.12

Two hundred sixty-nine participants (43%) indicated they did not know if, or how often, their spirometer should be calibrated. Only 85 participants (13%) indicated that they used the recommended Global Lung Initiative (GLI) reference values for adults, and 70 participants (11%) that they used the recommended GLI reference values for children. 308 (49%) and 386 participants (61%) did not know which reference values were reported by their spirometer for adults and children, respectively.

Participants evaluated the survey as useful (average 74 points on a 0–100 scale, standard deviation (SD) 24 points) and would like more case-based surveys concerning use of spirometry in the future (average 91 points, SD 17 points).

## Discussion

We investigated how GPs in Norway would interpret a case history and spirometry recordings of a patient with COPD, as well as their knowledge about their own spirometer. Most GPs were able to recognize a non-significant reversibility test, and 81% correctly identified COPD as the most likely diagnosis. Less than 50% of GPs, however, were able to correctly identify the spirometric parameters used to diagnose COPD and to grade the airway obstruction. Many were unaware of which type of spirometer and reference values were in use in their practice.

### Strengths and limitations

An important strength of our study is the broad recruitment; the invitation was distributed through the membership records of the Norwegian GP Association. While probably not 100% complete, this is the most updated source of e-mail addresses for GPs in Norway. Still, the low participation rate is a weakness of our study, limiting our ability to draw general conclusions about spirometry in general practice. Since there was self-selection of GPs into the survey, there is reason to believe that participating GPs, on average, were more interested in respiratory medicine than non-participants. Consequently, when we find knowledge gaps and problem areas, we probably underestimate rather than overestimate the challenges in this area for the GP population at large. Also, the design of a cross-sectional survey means we cannot draw inferences about cause and effect. Finally, as respondents were anonymous, a follow-up study to asses learning effects of the survey cannot be performed, leaving us only to speculate whether short surveys can be useful tools in continuous medical education.

Current Norwegian COPD guidelines [6] are in line with the GOLD recommendations, using post-bronchodilator fixed ratio of *FEV*_*1*_*/FVC < 0.7* to spirometrically define airway obstruction [7]. The fixed ratio is a choice of diagnostic simplicity and may lead to misclassifications, particularly overdiagnosis of COPD in the elderly, as the *FEV*_*1*_*/FVC ratio* decreases with age [10]. A more accurate definition of airway obstruction is *FEV*_*1*_*/FVC ratio* below the lower 5th percentile observed in a healthy, relevant population. Modern spirometers report this lower limit of normal (LLN) as z-scores, as does a frequently used Norwegian web-based decision support tool in COPD (www.kolskalkulator.no). We did not include this parameter in our survey, which may be added in future surveys.

### Comparison to previous findings

Prevalence of COPD varies greatly between studies, probably due to differences between populations and diagnostic criteria used [11]. Both over- and under-diagnosis of COPD likely happen in parallel. Over-diagnosis of COPD is largely attributed to diagnosing without performing the required spirometry [11]. In a multi-center study from 20 countries, more than 60% of patients who reported that they had been diagnosed with COPD, did not have airflow obstruction on spirometry [12]. In a Norwegian study, 26% of patients with a medical record of COPD, did not fulfill the spirometric criteria [2]. Similar findings were reported in a UK study [3]. Underdiagnosing on the other hand, is mainly attributed to insufficient use of spirometry and missed diagnostic opportunities [11]. A retrospective UK study of almost 40,000 patients with COPD, based on electronic medical records from primary care, found that opportunities for diagnosis were missed in 85% of the patients in the 5 years immediately preceding the diagnosis [13]. A Danish study found that opportunistic screening of 6700 at-risk patients in primary care identified that almost 18% had COPD [14]. In our survey, a premise was that everyone requested a spirometry for our at-risk patient. Therefore, we could not estimate potential under- or over-diagnosis directly. However, our case history and spirometry recordings were designed to illustrate a textbook example of a COPD patient. In short, our results showed that the knowledge on use and interpretation of spirometry in Norwegian general practice was suboptimal.

In Sweden, 250 GPs participated in a questionnaire-based study to investigate knowledge about diagnosis and treatment of COPD in general practice [4]. Spirometry was frequently used, but only 23% of GPs correctly interpreted the spirometry on the basis of postbronchodilator parameters [4]. Our results were similar; less than 50% of GPs correctly identified spirometric parameters used to 1) diagnose COPD, and to 2) grade airway obstruction. Only 143 of 630 (23%) correctly identified both parameters.

### Interpretation and implications

In our survey, we observed that requesting more spirometries during a regular week, and serving more patients, were factors associated with a higher chance of recognizing the correct diagnosis. This could be an indication that requesting more spirometries improves interpretation skills or that GPs already competent in the use of spirometry request more spirometries. We can similarly speculate that serving more patients is associated with more experience and practice in this field, but we are not able to explore these matters further. There were no associations between number of spirometries requested or patients served and the other outcomes we examined, which, in addition to the self-selection of GPs into the study, means these observations must be interpreted with caution.

Efficient continuing medical educations on complex health issues can be challenging in general practice. As a follow up to the Swedish study [4], primary care centers were later randomized to compare the impacts of two types of continuing medical education designed to improve GPs knowledge of COPD, either traditional lectures or a case-based method, both consisting of two 2-h sessions within 3 months. Compared with a reference group, both interventions gave only marginal improvements in the GPs knowledge measured after 12 months [15]. Others have found at least short-term positive effects of educational interventions when used in combination (short lectures, case discussions, spirometry workshops, inhalation demonstrations) [16, 17]. Our survey is now available online as a “mini-course” of spirometry in COPD, an easily accessible resource for GPs in need of a quick repetition. The participants evaluated our survey as useful and said they would like more case-based surveys concerning the use of spirometry in the future. Thus, to offer an online repository of “mini-courses” in spirometry is a goal. Since respondents undertook the survey anonymously, a rigorous and long-term investigation of any learning effects of such a survey is not possible at present.

Spirometry recordings contain a large number of parameters that are not necessary for correct interpretation in general practice. Our results confirm that there is substantial confusion among GPs on which parameters to use. Therefore, more user-friendly spirometry software which displays and highlights important parameters and suppresses parameters of less importance, could perhaps also improve spirometry interpretation in general practice.

## Conclusion

We conducted a web-based survey, now available online as a “mini-course”, and identified knowledge gaps and problem areas in requesting and interpreting spirometry in Norwegian general practice. Whether such short online courses may be a useful resource to help improve lung disorder management in general practice, may be a future topic of investigation.

## Supplementary Information

**Additional file 1.** Survey translated into English.

## Data Availability

The dataset used during the current study is available from the corresponding author on reasonable request.

## Abbreviations

COPD: Chronic Obstructive Pulmonary Disease
FEV_1_: Forced Expiratory Volume in one second
FVC: Forced Vital Capacity
GOLD: Global Initiative for Chronic Obstructive Lung Disease
GP: General practitioner
