# Supplementary material for: Spirometry in chronic obstructive pulmonary disease in Norwegian general practice
Source: BMC Fam Pract. 2020 Nov 18;21:235. doi: 10.1186/s12875-020-01310-x (PMC7677845; doi:10.1186/s12875-020-01310-x)
Supplement: Supplementary file 1 — Additional file 1. Survey translated into English. [file 12875_2020_1310_MOESM1_ESM.pdf]

Spirometry 2019

This short survey has been prepared by Noklus in collaboration with the Norwegian GP Association and the Norwegian Respiratory Society, targeting primarily general practitioners using spirometry in diagnosis and follow up of patients. The aim is to highlight and revise a common problem and provide support and education in interpretation of spirometric recordings. Participation is voluntary and results may be used in research and published.

* 1. Is this the first time you respond to the survey?

- Yes
- No

* 2. What is your main professional position?

- General practitioner
- Medical doctor, other
- Co-worker at general practitioner office
- Other (please specify)

* 3. Which type of spirometer do you use?

- Welch-Allyn
- Spirare
- Don't know
- Other (please specify)

4. A previously healthy man in the age group 50-59 years presents with complaints of progressive breathlessness on physical exertion over the past year. He has never had atopic symptoms, asthma or allergy, and there is no family history of lung- or upper airway disorders. He started smoking in his late teens but quit four years ago. You request a spirometry. Do you include a reversibility test or not?

- Include reversibility test
- Don't include reversibility test

The correct answer is to include a reversibility test.

When suspecting a pulmonary obstructive disorder, it is important to verify whether the condition is reversible or not. Significant reversibility can strengthen the suspicion of asthma, and post-bronchodilator spirometric values are used to diagnose and grade chronic obstructive pulmonary disorders (COPD). Requesting a spirometry including a reversibility test is therefore necessary.

1. *Kols. Nasjonal faglig retningslinje og veileder for forebygging, diagnostisering og oppfølging. Helsedirektoratet, November 2012*

5. Below, you see the spirometry recording. Is there significant reversibility?

- Yes
- No
- Not possible to evaluate
- Don't know

Testresultater:

FEV1%Pred: 48,6 %
FEV1%: 59,4%
Forbedring: FVC: 5,9%, FEV1: -5,9% ((Post - Pre) / Pre) * 100
Ikke vesentlig BD-respons

FEV1 Pre-/postvar: 79 ml (4,6 %) / 36 ml (2,2 %)
FVC Pre-/postvar: 106 ml (3,7 %) / 39 ml (1,3 %)
ATS-reproduserbarhet:
Pre: MET ()
Post: MET ()

Testtolkning: UBEKREFTET RAPPORT

Testkommentar:

Pre: FVC= 2,88L FEV1= 1,71L
FEV1%= 59,4% [1,71/2,88 FEV1/Max(FVC, FIVC, SVC)]

Post: FVC= 3,1L (5,9%); FEV1= 1,6L (-5,9%);
FEV1%= 52,7% [1,61/3,05 FEV1/Max(FVC, FIVC, SVC)] (-11,2%)

Parameter	Enh.	Forv (LLN)	Beste forsøk Pre	%Pred (z)	Post	%Pred (z)	%Endre
**FVC**	(L)	4,54 (3,47)	2,88*	63,5% (-2,55)	3,05*	67,2% (-2,29)	**5,9% (0,17)**
**FEV1**	(L)	3,52 (2,66)	1,71*	48,6% (-3,31)	1,61*	45,7% (-3,48)	**-5,9% (-0,10)**
**FEV1%**	(%)	77,7 (65,7)	59,4*	76,4% (-2,41)	52,7*	67,9% (-3,13)	**-11,2% (-6,6)**
**PEF**	(L/s)	-	4,76	-	4,84	-	**1,6% (0,08)**
**ATS**	-	-		-		-	-

Postmedikasjon: Ventolin, 400

(*) Betyr under LLN

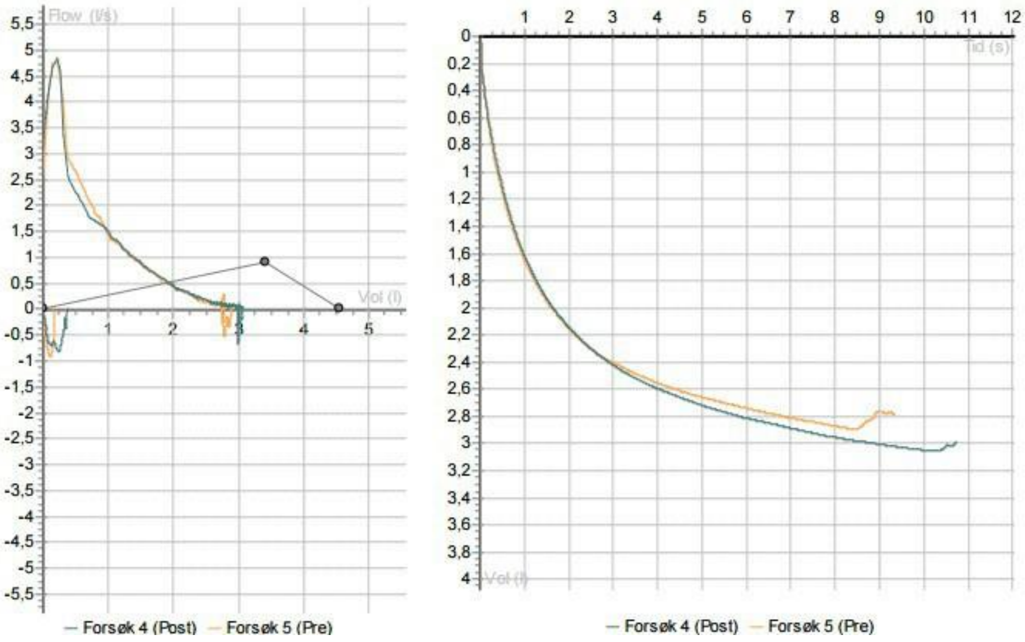

The correct answer is no.

FEV_1 should increase by at least 12% and 200 mL to conclude there is significant reversibility (1). In this patient, there is a decrease of 6% in FEV_1 after bronchodilation.

(1) Global intiative for asthma (GINA). Global strategy for asthma management and prevention (Updated 2019). https://ginasthma.org/

6. What is the most likely diagnosis?

- No pulmonary disorder
- Asthma
- Restrictive pulmonary disorder
- COPD
- Other
- Not possible to evaluate
- Don't know

The correct answer is COPD.

7. Which parameter(s) did you use to diagnose COPD?

- FVC
- FVC in percent of predicted (% Pred)
- FEV_1
- FEV_1 in percent of predicted (% Pred)
- FEV_1/FVC ratio (FEV1%)
- FEV_1/FVC ratio in percent of predicted (% Pred)

The correct answer is FEV_1/FVC ratio (FEV1%)

The spirometric criterion for diagnosing COPD is a post-bronchodilator FEV_1/FVC value < 0,70 (1,2). The Global Initiative for Chronic Obstructive Lung Disorder (GOLD) has chosen an age-independent diagnostic criterion to simplify the diagnostic criteria, even if FEV_1/FVC decreases with age. The spirometric criterion therefore contributes to overdiagnosis of COPD in elderly and underdiagnosing of COPD in younger patients. For patients younger than 50 years, COPD should be suspected at FEV_1/FVC < 0,75, while in elderly non-smokers, diagnosing COPD may be delayed until FEV_1/FVC < 0,65.

This patient has a post-bronchodilator FEV_1/FVC value of 0.53 (52.7%) and therefore fulfils the spirometric criterion for COPD.

(1) Global Initiative for chronic obstructive lung disease (GOLD). Global strategy for the diagnosis, management, and prevention of chronic obstructive pulmonary disease (Updated 2019). https://goldcopd.org/
(2) Kols. Nasjonal faglig retningslinje og veileder for forebygging, diagnostisering og oppfølging. Helsedirektoratet, November 2012.

8. Which parameter(s) did you use to grade the severity of COPD?

- FVC
- FVC in percent of predicted (% Pred)
- FEV_1
- FEV_1 in percent of predicted (% Pred)
- FEV_1/FVC ratio (FEV1%)
- FEV_1/FVC ratio in percent of predicted (% Pred)

The correct answer is FEV_1 in percent of predicted.

The GOLD criteria (1) use FEV_1 in percent of predicted to grade the severity of COPD:

Stage I: Mild - FEV_1 ≥ 80% of predicted

Stage II: Moderate - FEV_1 50–79% of predicted

Stage III: Severe - FEV_1 30-49% of predicted

Stage IV: Very severe, FEV_1 < 30% of predicted

This patient has FEV_1 in percent of predicted of 46-49, and thus has severe COPD.

(1) Global intiative for chronic obstructive lung disease (GOLD). Global strategy for the diagnosis, management, and prevention of chronic obstructive pulmonary disease (Updated 2019). http://www.goldcopd.org/

9. Which region of Norway do you work in?

10. How many patients do you serve?

- < 500
- 500-999
- 1000-1499
- 1500-1999
- 2000+

11. How many spirometries do you request during a regular week?

- None
- 1-4
- 5-9
- 10+
- Not relevant

12. How many spirometries in total are requested at your office during a regular week?

- None
- 1-4
- 5-9
- 10+
- Don't know
- Not relevant

13. How often is your spirometer calibrated?

- Daily
- Weekly
- Monthly
- Rarely/never
- My spirometer does not require calibration
- Don't know

14. Which reference values are used for adults?

The Global Lung Initiative (GLI) are recommended for adults and children.

- GLI (Global Lung Initiative)
- Gulsvik
- Langhammer
- Johannessen
- Other
- Don't know
- Not relevant

15. Which reference values are used for children?

The Global Lung Initiative (GLI) are recommended for adults and children.

- GLI (Global Lung Initiative)
- Zapletal
- Polgar/Solymar
- Other
- Don't know
- Not relevant

16. Did you find this survey useful? (Scale 0-100)

17. Would you like Noklus, the Norwegian GP Association and the Norwegian Respiratory Society to conduct more surveys regarding the use of spirometry? (Scale 0-100)

18. If yes, do you have suggestions on topic?

Thank you for your participation!
